# Mechanically Activated Calcium Channel PIEZO1 Modulates Radiation-Induced Epithelial-Mesenchymal Transition by Forming a Positive Feedback With TGF-β1

**DOI:** 10.3389/fmolb.2021.725275

**Published:** 2021-10-13

**Authors:** Jia-Qi Huang, Hao Zhang, Xue-Wei Guo, Yan Lu, Si-Nian Wang, Bo Cheng, Su-He Dong, Xiao-Li Lyu, Feng-Sheng Li, Yong-Wang Li

**Affiliations:** ^1^ The Postgraduate Training Base of Jinzhou Medical University (The PLA Rocket Force Characteristic Medical Center), Beijing, China; ^2^ Department of Anesthesiology, The PLA Rocket Force Characteristic Medical Center, Beijing, China; ^3^ Department of Neurology, The PLA Rocket Force Characteristic Medical Center, Beijing, China; ^4^ Department of Nuclear Radiation Injury and Monitoring, The PLA Rocket Force Characteristic Medical Center, Beijing, China; ^5^ Pathology Department, The PLA Rocket Force Characteristic Medical Center, Beijing, China; ^6^ Medical College of Soochow University, Suzhou, China; ^7^ The Third people’s Hospital of Longgang District Shenzhen, Shenzhen, China

**Keywords:** PIEZO1, epithelial-mesenchymal transition, TGF-β1, HIF-1α, C/EBPβ

## Abstract

TGF-β-centered epithelial-mesenchymal transition (EMT) is a key process involved in radiation-induced pulmonary injury (RIPI) and pulmonary fibrosis. PIEZO1, a mechanosensitive calcium channel, is expressed in myeloid cell and has been found to play an important role in bleomycin-induced pulmonary fibrosis. Whether PIEZO1 is related with radiation-induced EMT remains elusive. Herein, we found that PIEZO1 is functional in rat primary type II epithelial cells and RLE-6TN cells. After irradiation, PIEZO1 expression was increased in rat lung alveolar type II epithelial cells and RLE-6TN cell line, which was accompanied with EMT changes evidenced by increased TGF-β1, N-cadherin, Vimentin, Fibronectin, and α-SMA expression and decreased E-cadherin expression. Addition of exogenous TGF-β1 further enhanced these phenomena *in vitro*. Knockdown of PIEZO1 partly reverses radiation-induced EMT *in vitro*. Mechanistically, we found that activation of PIEZO1 could upregulate TGF-β1 expression and promote EMT through Ca^2+^/HIF-1α signaling. Knockdown of HIF-1α partly reverses enhanced TGF-β1 expression caused by radiation. Meanwhile, the expression of PIEZO1 was up-regulated after TGF-β1 co-culture, and the mechanism could be traced to the inhibition of transcription factor C/EBPβ expression by TGF-β1. Irradiation also caused a decrease in C/EBPβ expression in RLE-6TN cells. Dual luciferase reporter assay and chromatin immunoprecipitation assay (ChIP) confirmed that C/EBPβ represses PIEZO1 expression by binding to the PIEZO1 promoter. Furthermore, overexpression of C/EBPβ by using the synonymous mutation to C/EBPβ siRNA could reverse siRNA-induced upregulation of PIEZO1. In summary, our research suggests a critical role of PIEZO1 signaling in radiation-induced EMT by forming positive feedback with TGF-β1.

## Introduction

The human lung is sensitive to ionizing radiation (Ding et al., 2013). Radiation-induced pulmonary injury (RIPI) can be seen not only in patients with chest tumors radiotherapy, but also in practitioners receiving long-term low-dose radiation (Dentesano et al., 2012; Kumar et al., 2013; Douguet and Honore, 2019; World Health Organization, 2020). Approximately 35% of lung and breast cancer patients will develop RIPI after receiving chest radiation therapy (Chen et al., 2019). RIPI was mainly divided into three stages, which are asymptomatic phase (He et al., 2019), radiation-induced pneumonitis (Hanania et al., 2019) and radiation-induced pulmonary fibrosis (Graves et al., 2010; Chen et al., 2019). When the disease progresses to the third stage, irreversible damage to the respiratory system occurs and the long-term quality of life was greatly impaired (Graves et al., 2010; Chen et al., 2019). Finding interventions to delay and reverse the development of RIPI remains a critical issue in current clinical practice.

Alveolar type II epithelial (AT II) cells play a key role in ionizing radiation-induced lung injury (Chen et al., 2019). After irradiation, the injured/damaged epithelial cells undergo epithelial-to-mesenchymal transition (EMT), which has long been confirmed to have a central role in lung fibrosis (Sisson et al., 2010). TGF-β1 represent the core regulators of EMT by forming complex network signaling pathways (J. Zhang et al., 2014). TGF-β1 signaling is modulated by hypoxia inducible factor-1α (HIF-1α). HIF-1α is considered essential for the malignant transformation of meloblastoma *via* TGF-β-dependent EMT (Yoshimoto et al., 2019). The novel HIF-1α inhibitor, 2-methoxyestradiol (2-ME) could efficiently inhibit radiation-induced lung fibrosis in a preclinical study (Nam et al., 2021). However, it is still not known whether HIF-1α also mediates radiation-induced EMT.

The activity of HIF-1α is elegantly regulated by calcium signaling (Azimi, 2018). As a mechanically sensitive Ca^2+^ channel, PIEZO1 is expressed in various lung cells including epithelial cells (bronchus and alveolus) and endothelial cells (Bhattacharya and Hough, 2019; Friedrich et al., 2019; Ridone et al., 2019). In AT II epithelial cells, mechanical stress during the respiratory cycle activates PIEZO1, causing Ca^2+^ influx, thereby releasing alveolar surfactant (Diem et al., 2020). A recent study has found that cyclical hydrostatic pressure can trigger an inflammatory response by activating PIEZO1/HIF-1α signaling in myeloid cells of the lung (Solis et al., 2019). However, whether there is a role of PIEZO1 signaling in radiation-induced EMT is not confirmed.

C/EBPβ belongs to a family of highly conserved transcription factors composed of multiple functional domains including a basic-leucine zipper at the C-terminus in juxtaposition of a basic domain that is responsible for DNA binding (Tolomeo and Grimaudo, 2020). The pathway activity of C/EBPβ can be regulated by TGF-β1. TGF-β was found to induce CTGF expression through the ERK/ADAM17/RSK1/C/EBPβ signaling pathway in human lung epithelial cells (Ou et al., 2020). Nonetheless, opposite findings have been reported. A study revealed that C/EBPβ expression was repressed by TGF-β1 through the canonical Smad3 pathway in NMuMG cells and miR-155-mediated repression C/EBPβ could sensitize cells to TGF-β1-induced EMT (Ramji and Foka, 2002). We searched the PROMO (prediction of transcription factor binding sites) and found a potential binding site of C/EBPβ in the PEIZO1 promoter locus (Messeguer et al., 2002). Thus, there is a possibility that TGF-β1 and PIEZO1 might interact to promote EMT after irradiation. To verify the aforementioned hypotheses, the current study is performed.

## Materials and Methods

### Cell Lines and Cell Culture

Rat alveolar type 2 epithelial cells (RLE-6TN) were purchased from Eallbio Life Sciences (Beijing, China, Cat NO: 06.0627). Primary rat alveolar epithelial type II (ATII) cells were bought from Procell, China (Cat NO: CP-R003). Both kinds of cells were cultured in Roswell Park Memorial Institute (RPMI) 1,640 Medium containing 10% fetal bovine serum (Gibco, United States), 100 U/ml penicillin and 100 mg/ml streptomycin (Gibco, United States). The cells were incubated in an incubator at 37°C with saturated humidity and 5% CO_2_. The culture medium was changed every other day.

### Animals and Ethics Statement

Adult male Sprague-Dawley rats were purchased from the SPF (Beijing) Biotechnology Co., Ltd. and kept in the animal room of PLA Rocket Force Characteristic Medical Center. The rats were housed at a controlled temperature of 24°C–26°C with 50 ± 5% relative humidity. Rats were fed with at 12:12 h light/dark cycle with free access to food and water. All experimental procedures were performed according to Guide for the Care and Use of Laboratory Animals (eighth edition, National Academies Press, Washington, DC, 2011) and approved by the local ethics review board.

### Irradiation of RLE-6TN Cells and Rats

Once cells reached >80% confluence, RLE-6TN cells or primary rat lung epithelial cells were irradiated with a single dose of 12 Gy X-rays. The radiation was performed using a 250 kVp orthovoltage machine with a dose rate of 1 Gy/min. Irradiations were performed at room temperature, while non-irradiated control cells were studied in parallel, at the same conditions. The timing of drug incubation and indicators evaluation were described later in the individual experiments.

For the irradiation of rat lungs, the rats were firstly anesthetized using 40 mg/kg of sodium pentobarbital. Then a 5 mm-thick lead block was used to shield the rest part of the animal except the whole thorax. The rats received a whole thorax dose of 12 Gy at a dose rate of 1 Gy/min. After irradiation, the rat lung tissues were fetched for immunohistochemistry and Western blotting examination of PIEZO1 protein expression. This dosage was selected according to our preliminary results and a previous study, which showed acute and long-lasting increase in the expression of TGF-β in lung tissue from AT II cells and fibroblasts following thoracic irradiation with 12 Gy (Rube et al., 2000). The experimental results were got 48 h after irradiation unless other time points were specified. For cell transfection studies, the specific siRNAs (si-PIEZO1, si-HIF-1α and si-C/EBPβ) were incubated from 24 h before irradiation until 48 h after irradiation.

### RT-qPCR

Total RNA was extracted from cells using the TRIzol Reagent (Sigma, United States), and mRNA was reverse transcribed using the PrimeScript RT reagent Kit with gDNA Eraser (TAKARA, Dalian, China). Real-time PCR was performed using the SYBR Green Taq Mix (TAKARA, Dalian, China) kit. The amplification conditions were as follows: 95°C for 30 s to pre-denaturation, 40 cycles at 95°C for 5 s to denaturation, and 60°C for 30 s to annealing and extension. The primers used were as follows**:** PIEZO1, Forward: 5′-CGG​ACA​GTG​AGG​AGG​AAG​AGG​AG-3′ and Reverse: 5′-CCT​GTT​CAC​GAC​GCT​GCC​TTA​G-3’; HIF-1α, Forward: 5′-ATG​GTG​CTA​ACA​GAT​GAT​GG-3′ and Reverse: 5′-TAG​TTC​AAA​CTG​AGT​CAA​CCC-3’; TGF-β1, Forward: 5′-GAC​CGC​AAC​AAC​GCA​ATC​TAT​GAC-3′ and Reverse: 5′-CTG​GCA​CTG​CTT​CCC​GAA​TGT​C-3’; C/EBPβ, Forward: 5′-GCT​GAG​CGA​CGA​GTA​CAA​GAT​GC-3′ and Reverse: 5′-CTT​GTG​CTG​CGT​CTC​CAG​GTT​G-3’; GAPDH, Forward: 5′-CAG​TGC​CAG​CCT​CGT​CTC​AT-3′ and Reverse: 5′-AGG​GGC​CAT​CCA​CAG​TCT​TC-3’. β-actin, Forward: 5′-TCA​GGT​CAT​CAC​TAT​CGG​CAA​T-3′ and Reverse: 5′- AAA​GAA​AGG​GTG​TAA​AAC​GCA-3’. The results were calculated and analyzed according to the 2^−ΔΔCt^ method with GAPDH or β-actin as the control.

### Western Blotting

Rat lung tissues or cells were lysed with RIPA lysis buffer. The concentration of protein was determined using a BCA Protein Assay kit (Beyotime, China). 20 μg of total protein were separated by 10% SDS-PAGE, followed by transfer to polyvinylidene fluoride membranes (Bio-Rad, United States). The membranes were blocked with 5% defatted milk in Tris Buffered saline Tween (TBST) for 1 h at room temperature and then incubated with antibody at 4°C overnight. Following three washings of the membrane in TBST (10 min each), a secondary antibody (1:5,000, Abcam, UK) was incubated for 1 h at room temperature followed by three washings (10 min each) with TBST. After chemiluminescence, the gray values of target bands and the internal reference (GAPDH, β-actin, β-tubulin or Vinculin) were quantified by ImageJ software.

The antibodies used in this experiment were as follows: anti- PIEZO1 (Abcam, United States; ab128245, 1:5); anti-HIF-1α (Cell Signaling Technology, United States; 14179, 1:1,000); anti-TGF-β1 (Cell Signaling Technology, United States; 3711, 1:1,000); anti-C/EBPβ (BioLegend, United States 606202, 1:1,000); anti-Fibronectin (Proteintech, United States 15613-1-AP, 1:1,000); anti-α-Smooth Muscle Actin (α-SMA) (Cell Signaling Technology, United States; 19245, 1:1,000); anti-CTGF (Abcam, United States; ab6992, 1:1,000); anti-E cadherin (Abcam, United States; ab1416, 1:1,000); anti-Vimentin (Cell Signaling Technology, United States; 3932, 1:1,000) anti-GAPDH (Proteintech, United States; 60004-1-Ig, 1:2,000); anti-Vinculin (Boster, United States; A30448,1:1,000); anti-β actin (Proteintech, United States; 60008-1-Ig, 1:1,000); anti-β-tubulin (Proteintech, United States; 10094-1-AP, 1:1,000).

### Transfection

Cells were cultured under standard conditions until the confluency reached about 60–80%. The small interfering RNAs (siRNAs) specifically targeting PIEZO1, HIF-1α and C/EBPβ were designed and produced by GenePharma (Suzhou, China), which were named as si-PIEZO1, si-HIF-1α and si-C/EBPβ respectively. RLE-6TN cells were transfected with 100 nM siRNAs by using Lipofectamine RNAi MAX (Invitrogen, United States). The culture medium was exchanged 24 h after transfection. The expression of mRNA was measured by RT-qPCR after 36 h of cultivation, and Western blotting was performed after 48 h of cultivation. The siRNA sequence was as follows: si-PIEZO1, 5′-CGG​CCA​ACA​UAA​AGA​ACA​UTT-3′; si-HIF-1α, 5′- GAT​GGA​AGC​ACT​AGA​CAA​A-3’; si-C/EBPβ, 5′- CGC​CTT​TAG​ACC​CAT​GGA​A-3’; si-NC, 5′- UUC​UCC​GAA​GUC​ACG​UTT-3’.

According to the targeting sequence (5′- GCC​GCC​TTT​AGA​CCC​ATG​GAA-3′) of si-C/EBPβ, a C/EBPβ cDNA carrying the corresponding synonymous mutation sequence (5′-GCT​GCT​TTC​AGG​CCT​ATG​GAG-3′) was cloned in the pcDNA3.1 expression vector. For plasmid transfection, 1 × 10^5^ RLE-6TN cells were seeded into 12-well plates 24 h before siRNA transfection. After 24 h siRNA transfection, the cells were transfected with 0.5 µg of plasmid. Transfections were performed using Lipofectamine 3,000 reagent (Invitrogen), and cells were collected 72 h after transfection. The plasmid was constructed by Obio (Obio, shanghai, China) and the transfection efficiency has been previously verified.

### Immunofluorescence

The cells were fixed with 4% PFA for 30 min and then permeabilized with 0.2% Triton X-100 for 10 min at room temperature. Thereafter, the cells were blocked with 3% normal goat serum at 37°C for 1 h and incubated with primary antibodies overnight at 4°C. After washing, CoraLite594-conjugated Goat Anti-Rabbit IgG (H + L) (Proteintech, United States; SA00013-4, 1:250) were added. The cells were further immersed in 40, 6-diamidino-2-phenylindole (DAPI, 1:500, Abcam, UK) for 5 min to visualize nuclei. A fluorescence microscope (Leica, Germany) was used to detect the expression and intracellular location of the PIEZO1 and HIF-1α protein. The antibodies used in this experiment were as follows: anti- PIEZO1 (Abcam, United States; ab128245, 1:250) and anti-HIF-1α (Novartis, United States; NB100-479, 1:100). Images were cropped and adjusted using ImageJ. The mean immunofluorescence intensities of images were calculated using ImageJ.

### Immunohistochemistry

Differently treated adult male SD rat lungs were removed, fixed in 4% paraformaldehyde (PFA), embedded in paraffin, and sectioned at 5 μm thickness for immunohistochemistry. Slices underwent xylene dewaxing, rehydration by a graded series of ethanol, washing with distilled water and PBS, and blocking for endogenous peroxidases through 15 min of incubation with 3% H_2_O_2_ in methanol. Antigen retrieval procedure was then undertaken on the slices *via* microwave processing in the sodium citrate buffer (0.01 M, pH 6.0). The slices were washed again with PBS and then incubated with 10% normal goat serum at 37°C for 30 min. Incubation of the slices lasted overnight at 4°C with the anti- PIEZO1 (Abcam, United States; ab128245, 1:100), anti-TGF-β1 (Cell Signaling Technology, United States; 3711, 1:100), anti-Fibronectin (Proteintech, United States; ab175430, 1:100), anti-α-Smooth Muscle Actin (α-SMA)(Cell Signaling Technology, United States; 19245, 1:1,000), and anti-CTGF (Abcam, United States; ab6992, 1:100). Then the slices washed with PBS were allowed to expose to 1% biotinylated secondary antibody goat anti-rabbit IgG (Boster, Wuhan, China) at 37°C for 1 h, followed by incubation with streptavidin-biotin complex (SABC) (Boster, Wuhan, China) at 37°C for 30 min. For visualizing immunoreaction, diaminobenzidine hydrochloride (DAB) (Boster, Wuhan, China) was used to immerse the slices. Immediately after a brown color staining was visualized, the slices were observed under a microscope and stopped through immersion in distilled water. The slices were then subjected to light counterstaining by hematoxylin, dehydration by ethanol, xylene clearing and then mounted. Images were taken using a microscope (Leica, Germany). Images were cropped and adjusted using ImageJ. The number of cells stained positive for the abovementioned antibodies under 400 X microcopy with were used for comparison among groups.

### Measurement of Intracellular Ca^2+^ Concentrations

Fluo4-AM (Dojindo, Kumamoto, Japan) was used to test the intracellular Ca^2+^concentration (Mochizuki et al., 2009). RLE-6TN cells were cultured under standard conditions until the confluency reached about 60–80%. The cells were incubated with Fluo4-AM at a working concentration of 5 µM for 30 min at 37°C. After washing the cells once with a Ca^2+^ free and Mg^2+^-free balanced salt solution, the cells were further incubated at 37°C for 20 min. Then the cells were digested with 0.25% trypsin and resuspended in balanced salt solution. The Fluo4-AM fluorescence signal was detected by flow cytometry at the excitation wavelength of 494 nm and the emission wavelength of 516 nm. Analysis of Ca^2+^ concentration was performed based on relative fluorescence intensity using the FlowJo software.

### Dual-Luciferase Reporter Assay

The PROMO (prediction of transcription factor binding sites) database was used to predict the binding site of PIEZO1 promoter to C/EBPβ. Then, wild type (WT) and mutant type (MUT) fragment sequences of PIEZO1 promoter (0–2.0 kb from the PIEZO1 transcription start site) were inserted between the Kpnl and HindIII sites on firefly luciferase pGL3 report vector (Promega, Madison, WI, United States). HEK-293 T cells were seeded in 96-well culture plates (5,000 cells/well). The cells were transiently co-transfected with the generated firefly luciferase vectors and either the C/EBPβ shRNA expression plasmid or a control, non-specific shRNA using Lipofectamine 2000 following the manufacturer’s instructions. After transfection, the dual-luciferase reporter assay system (Promega) was used to detect relative luciferase activity.

### Chromatin Immunoprecipitation

RLE-6TN cells were incubated for 10 min at a final concentration of 1% formaldehyde in a 37°C incubator. Cross-linking was terminated by adding 125 mM glycine. After rinse, the cells were harvested and sonicated to generate DNA fragments at the length of 200–1000 bp. After that, centrifugation was performed at 12,000 rpm for 10 min at 4°C. The supernatant was then diluted with IP buffer (0.01% SDS, 1.1% Trition-X 100, 1.2 mM EDTA, 16.7 mM Tris-Cl, pH 8.1,167 mM NaCl) plus protease inhibitor for 1:10 dilution of the cross-linked DNA/protein. The samples were then precleared with 60 μl of protein A + G agarose (Upstate Biotech, Charlottesville, VA) for 1 h at 4°C. Approximately 1/10 of the sample is taken as an input control and the rest is immunoprecipitated with 1 μg of anti-C/EBPβ (Upstate Biotech, Charlottesville, VA) antibody or control IgG (negative control) overnight at 4°C with agitation. Chromatin/antibody complexes were then collected with Protein A + G agarose, followed by washing and elution according to the manufacturer’s procedures. DNA is purified from the input chromatin and immunoprecipitation eluate by reversal of crosslinking and purification with 200 mM NaCl at 55°C. The purified DNA was then subjected to real-time PCR. The PIEZO1-1 promoter fragment (247 bp) was amplified using the following primer pair PIEZO1-1F (5′-GCT​AAA​TCC​CCA​ACC​CTC​C) and PIEZO1-1R (5′- TAC​CCT​GCA​CAA​CAG​ATA​GTT​ACA​T). The PIEZO1-2 promoter fragment (198 bp) was amplified by primers PIEZO1-2F (5′- CTG​AAT​CCC​TGT​CTA​CTC​TGG​C) and PIEZO1-2R (5′- TCT​GGT​TCA​CCT​TTC​TCC​CTC).

### Statistical Analyses

GraphPad software 8.0 (GraphPad Software, Inc, La Jolla, CA, United States) were used to perform statistical analyses. Data are presented as the means ± standard errored of means (SEM). Differences among groups were evaluated by one-way analysis of variance (ANOVA) followed by post hoc Tukey’s pairwise comparison. Each experiment was repeated as three independent experiments unless specified. *p* < 0.05 was considered statistically significant.

## Results

### PIEZO1 Expression Was Confirmed in Rat Lung at II Cells and Was Upregulated After Irradiation

**FIGURE 1:**
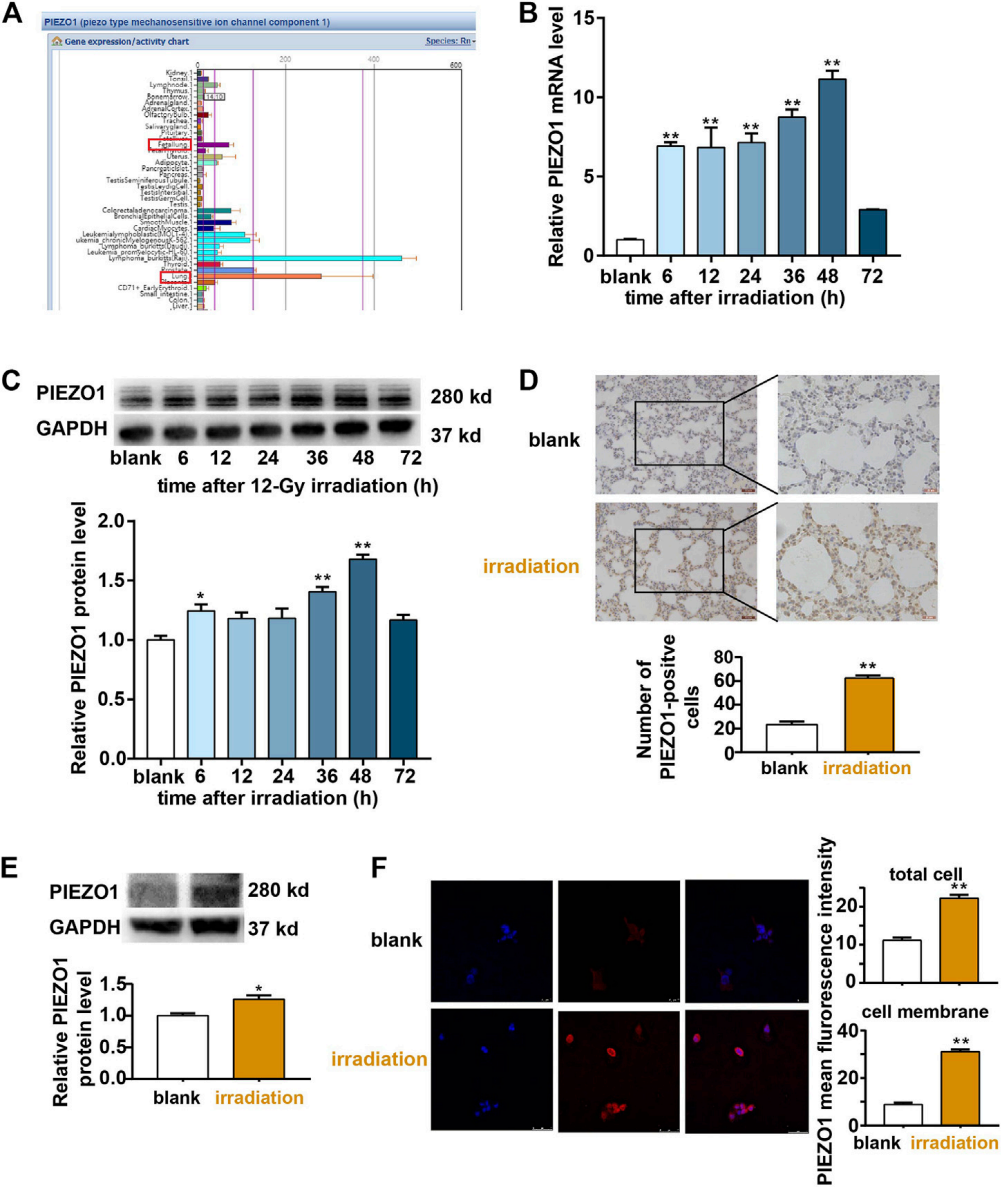
PIEZO1 expression was upregulated after irradiation (IR). **(A)** BioGPS database demonstrated that PIEZO1 is widely expressed in various kinds of lung. **(B)** PIEZO1 mRNA expression was increased in RLE-6TN cells after irradiation. **(C)** Representative Western blotting images and histograms showed that PIEZO1 protein expression was increased 48 h after irradiation (IR). **(D)** Immunohistochemistry studies revealed that PIEZO1 expression was upregulated in rat lung epithelial cells 48 h after irradiation. The cell nucleus was stained with hematoxylin (blue) and PIEZO1 protein was stained using specific antibody with the DAB nickel method (dark brown). **(E)** Western blotting showed PIEZO1 protein expression in rat lung tissue expression was increased 48 h after irradiation **(F)** Immunofluorescence staining showed that PIEZO1 expression was upregulated in the cell membrane and total part of RLE-6TN cells 48 h after irradiation. Nuclei were stained with DAPI (blue). Data are presented with the means ± SEMs (n = 3). *, *p* < 0.05; and **, *p* < 0.01.

The search of BioGPS database revealed that PIEZO1 was widely expressed in various kinds of lung cells including AT II cells (Walker et al., 2004; Wu et al., 2009; Wu et al., 2013; Wu et al., 2016)(Figure 1A). After 12-Gy irradiation, increased expression of PIEZO1 mRNA (Figure 1B) and protein (Figure 1C) were found in RLE-6TN cells 48 h later compared to those of the control group respectively. The increased PIEZO1 protein expression as also observed in lung tissues in rats receiving whole chest 12-Gy irradiation by immunohistochemistry (Figure 1D) and Western blotting (Figure 1E). Immunofluorescence confirmed that PIEZO1 was expressed in the cell membrane, cytoplasm and nucleus of the RLE-6TN cells (Figure 1F). After irradiation, the cell membrane expression of PIEZO1 protein was significantly increased compared with control (Figure 1F). Based on these results, the following experiments were all performed 48 h post irradiation.

### PIEZO1 Participated in Radiation-Induced Epithelial-to-Mesenchymal Transition

**FIGURE 2:**
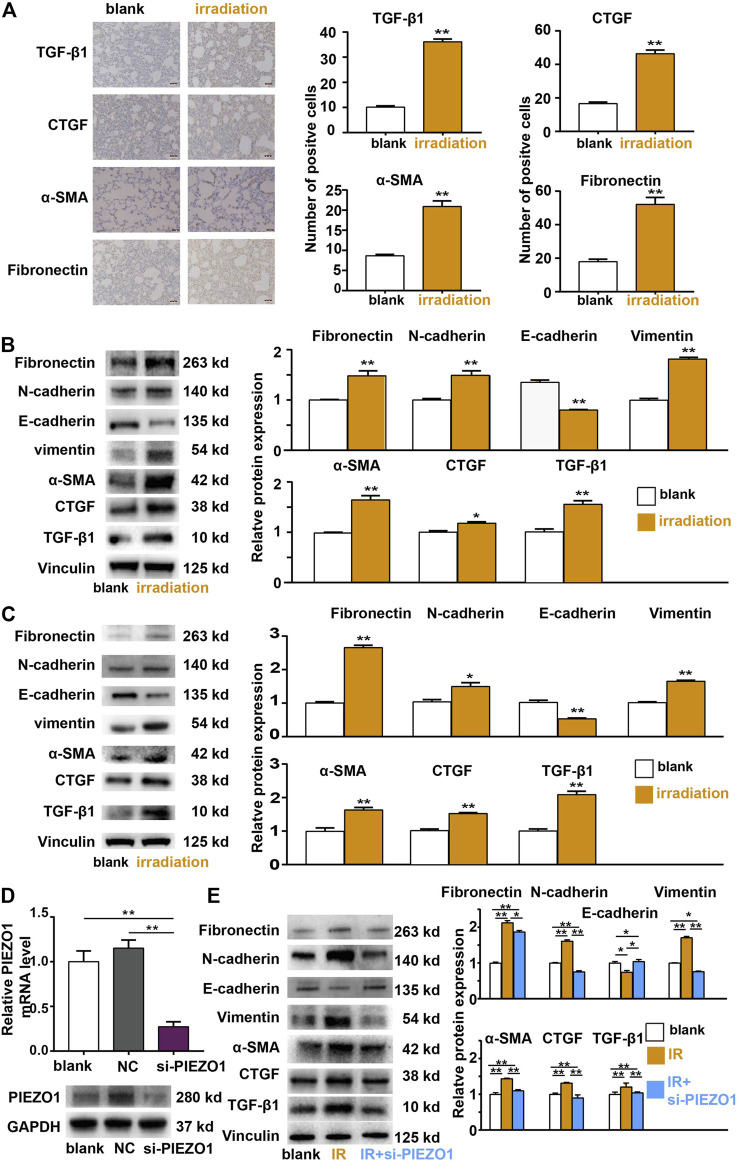
Epithelial-mesenchymal transition (EMT) occurred after irradiation (IR) and is mediated by PIEZO1 signaling. **(A)** Immunohistochemical analyses found TGF-β1, α-SMA, CTGF, and Fibronectin expression was increased 48 h after IR. **(B,C)** Western blotting results of rat lung tissues **(B)** and RLE-6TN cells **(C)** showed that the expression of EMT-related markers N-cadherin, Vimentin, TGF-β1, α-SMA, CTGF, and Fibronectin were up-regulated and E-cadherin was down-regulated compared to those of the control group 48 h after IR. **(D)** PIEZO1 mRNA and protein expression were successful inhibited by specific siRNA to PIEZO1 (si-PIEZO1) compared with blank and negative control (NC). **(E)** Representative Western blotting images and histograms showing that IR-induced EMT-related protein expression changes could be partly reversed by PIEZO1 knockdown. Note: The Western blotting images have been subjected to cropping and stitching inresponse to the reviewers’ comments. The original figures can be seen in the supplemental materials. Data are presented with the means ± SEMs (n = 3). *, *p* < 0.05; and **, *p* < 0.01.

Both rat lungs and RLE-6TN cells were subjected to 12 Gy radiation. Immunohistochemistry studies found that the expression of EMT-related markers including TGF-β1, Fibronectin, CTGF, and α-SMA was increased after irradiation (Figure 2A). The Western blotting results of rat lung tissues (Figure 2B) and RLE-6TN cells (Figure 2C) also showed that the expression of EMT-related markers including TGF-β1, Fibronectin, CTGF, α-SMA, N-cadherin and Vimentin was up-regulated and E-cadherin was decreased compared to those of the control group, respectively. These results suggests that 12 Gy radiation is sufficient to cause the EMT of rat lung AT II cells.

We then investigated whether radiation-induced EMT is relied on PIEZO1 pathway activity modulation. When PEIZO1 mRNA and protein expression were successfully inhibited by specific siRNA (incubated from 24 h before irradiation until 48 h after irradiation) to PIEZO1 (Figure 2D), IR-induced increase in expression of TGF-β1, α-SMA, CTGF, Fibronectin and Vimentin protein and decrease in E-cadherin protein expression were partly rescued by PIEZO1 knockdown (Figure 2E).

### PIEZO1 Regulates the Expression of TGF-β1 Through HIF-1α

**FIGURE 3:**
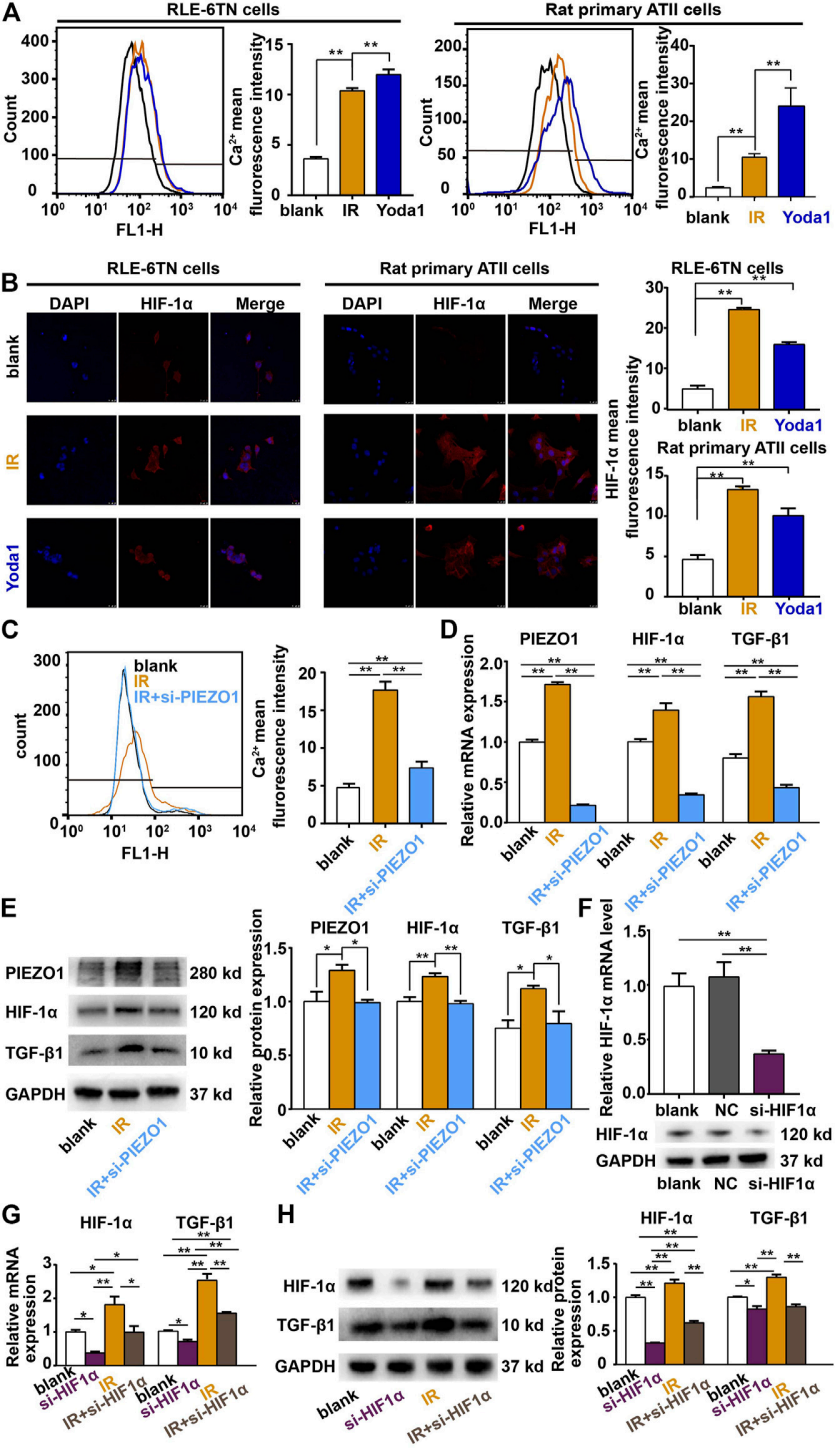
PIEZO1 modulates epithelial-mesenchymal transition *via* Ca^2+^/HIF-1α signaling. **(A,B)** Irradiation (IR) and the selective PIEZO1 activator Yoda1 increased calcium influx in RLE-6TN cells and primary rat lung epithelial cells. **(B)** Immunofluorescence staining showed that HIF-1α expression was upregulated by Yoda1 and IR. Nuclei were stained with DAPI (blue). **(C)** Flow cytometry measurement of intracellular Ca^2+^ concentrations found that IR-induced calcium influx was partly blocked by PIEZO1 knockdown. **(D)** qPCR results found that increased HIF-1α and TGF-β1 expression after IR was blocked by PIEZO1 knockdown. **(E)** Representative Western blotting images and histograms showing IR-induced PIEZO1, HIF-1α and TGF-β1 protein expression were blocked by PIEZO1 knockdown. Note: The Western blotting images have been subjected to cropping and stitching inresponse to the reviewers’ comments. The original figures can be seen in the supplemental materials. **(F)** HIF-1α mRNA and protein expression were successful inhibited by specific siRNA to HIF-1α (si-HIF-1α). **(G,H)** The enhanced expression of TGF-β1 mRNA **(G)** and protein **(H)** after IR was significantly reduced when HIF-1α expression was inhibited by si-HIF-1α. Data are presented with the means ± SEMs (n = 3). *, *p* < 0.05; and **, *p* < 0.01.

PIEZO1 belongs to calcium-permissible mechanosensitive channel. HIF-1α is a downstreaming effector of Ca^2+^ influx. We verified that PIEZO1 forms a functional ion channel in RLE-6TN cells and primary rat lung epithelial cells. In both kinds of cells, the use of selective PIEZO1 activator Yoda1 (2.5 for 48 h) or irradiation could increase calcium influx (Figure 3A) and downstreaming HIF1α expression (Figure 3B). Furthermore, PIEZO1 knockdown using the specific siRNA (Figure 2D) partly reverse the rise in intracellular calcium concentration caused by irradiation (Figure 3C). qPCR (Figure 3D) and Western blotting (Figure 3E) showed that the expression of HIF-1α mRNA and protein was further decreased with the knockdown of PIEZO1 compared to the irradiated group alone.

TGF-β1 plays a core role in EMT. A decrease in TGF-β1 mRNA and protein expression was found after PIEZO1 knockdown compared with irradiation alone. We further knocked down HIF-1α in RLE-6TN cells (Figure 3F) and found that under both the control and irradiation circumstances, the expression of TGF-β1 mRNA (Figure 3G) and protein (Figure 3H) was significantly reduced when HIF-1α expression was inhibited. Taken together, these results suggests that PIEZO1 acts through Ca^2+^/HIF-1α signaling to modulate the expression of TGF-β1.

### TGF-β1 Positive Feedback Causes Increased Expression of PIEZO1 *via* Transcription Factor C/EBPβ

**FIGURE 4:**
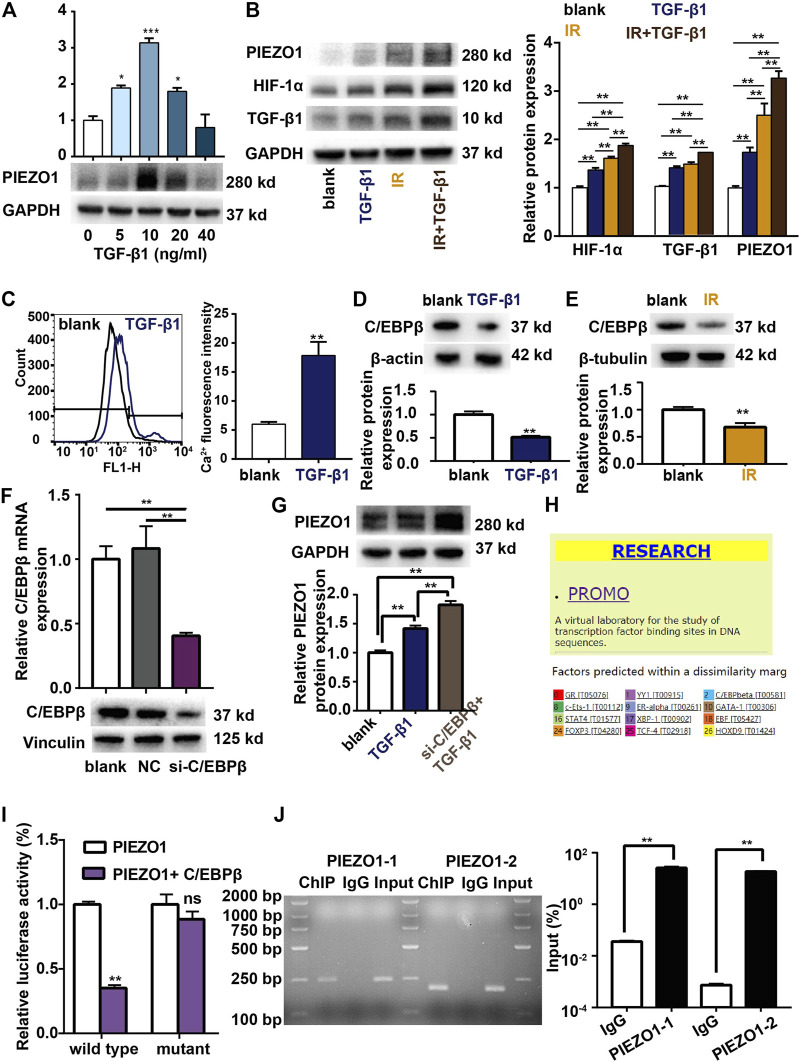
TGF-β1 promotes PIEZO1 expression through C/EBPβ. **(A)** TGF-β1 dose-dependently induces PIEZO1 mRNA and protein expression. **(B)** PIEZO1, HIF-1α and TGF-β1 protein expression were futher enhanced by addition of TGF-β1 to cells subjectd to irradiation (IR). **(C)** Intracellular Ca^2+^ concentration was increased after TGF-β1 co-culture. **(D)** TGF-β1 inhibits C/EBPβ protein expression. **(E)** C/EBPβ protein expression was decreased after IR. **(F)** C/EBPβ mRNA and protein expression were successful inhibited by specific siRNA to C/EBPβ. **(G)** Compared to TGF-β1 co-culture alone, PIEZO1 protein was further increased when C/EBPβ expression was inhibited by siRNA targeting C/EBPβ (si-C/EBPβ). **(H)** The PROMO (prediction of transcription factor binding sites) database indicates a potential binding site of C/EBPβ in the PEIZO1 promoter locus. **(I)** Dual-luciferase reporter assay suggests that C/EBPβ binds to PIEZO1 gene promoter. **(J)** Chromatin immunoprecipitation (ChIP) reporter gene analyses found two binding sites of C/EBPβ in the upstream region of the PIEZO1 promoter. Data are presented with the means ± SEMs (n = 3). *, *p* < 0.05; and **, *p* < 0.01.

Previous findings have revealed several positive feedback networks of TGF-β signaling during fibrosis (K. Zhang et al., 2017). We further observed the potential positive feedback effects of TGF-β1 on PIEZO1 expression. PCR and Western blotting showed that 5–40 ng/ml of TGF-β1 incubation can significantly increase PIEZO1 mRNA and protein expression 48 h thereafter (Figure 4A). Compared to blank control, exogenous TGF-β1 also significantly increased the protein levels of HIF-1α and TGF-β1 (Figure 4B). Calcium influx was increased after TGF-β1 co-culture compared with blank (Figure 4C). Compared with irradiation alone, incubation of irradiated cells with TGF-β1 futher increased the expression of PIEZO1, HIF-1α and TGF-β1 (Figure 4B).

TGF-β1 co-culture down-regulates C/EBPβ expression compared with control (Figure 4D). Similarly, we found that C/EBPβ expression is inhibited after irradiation alone (Figure 4E). When C/EBPβ was inhibited by siRNA transfection (Figure 4F), the expression of PIEZO1 protein was further increased compared to TGF-β1 co-culture alone (Figure 4G). As the PROMO (prediction of transcription factor binding sites) database indicates a potential binding site of C/EBPβ in the PEIZO1 promoter locus (Figure 4H), we confirmed the presumed association of C/EBPβ factors with the PIEZO1-promoter by dual-luciferase reporter assay and chromatin immunoprecipitation (ChIP). The result showed that compared with the control group, the luciferase activity of C/EBPβ and PIEZO1 promoter co-transfected cells was lower (Figure 4I), while the luciferase activity of C/EBPβ and PIEZO1 promoter mutant co-transfected cells showed no significant difference (Figure 4I). For the ChIP experiments, following immunoprecipitation using C/EBPβ antibodies, DNA was recovered and subjected to PCR analysis using oligonucleotide primers flanking the two predicted binding cites of the PIEZO1 gene promoter. ChIP results shows the binding of the C/EBPβ factors to two different PIEZO1-promoter regions. RT-PCR showed that C/EBPβ was able to bind to PIEZO1 as a transcription factor (Figure 4J).

**FIGURE 5:**
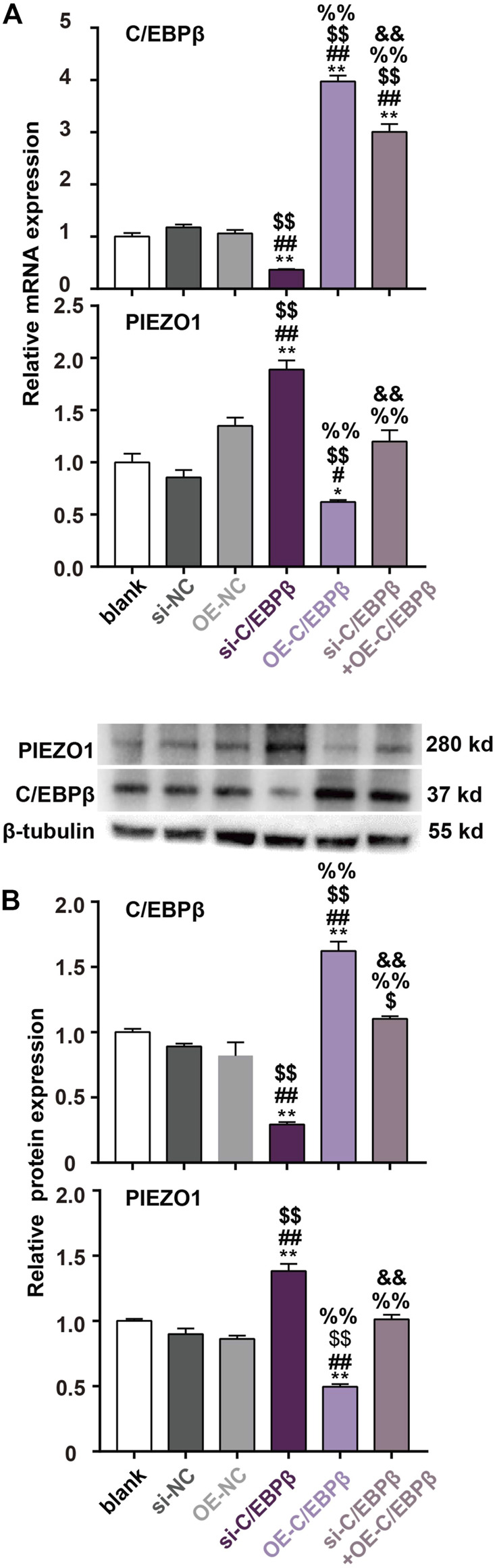
siRNA-resistant C/EBPβ plasmid transfection reverse the enhanced PIEZO1 expression by C/EBPβ small interfering RNA (si-C/EBPβ). **(A)** qPCR results showed that the changes of PIEZO1 and C/EBPβ mRNA levels after transfection with siRNA-resistant C/EBPβ plasmids (OE). **(B)** Representative Western blotting images and histograms showed that the expression changes of C/EBPβ and PIEZO1 protein after transfection with siRNA-resistant C/EBPβ plasmids. Data are presented with the means ± SEMs (n = 3). **, *p* < 0.01 *vs* control; ^##^, *p* < 0.01 *vs.* si-NC (negative control of siRNA); ^$$^, *p* < 0.01 *vs* OE-NC (negative control of siRNA-resistant C/EBPβ plasmid); ^%%^, *p* < 0.01 *vs* si-C/EBPβ; ^&&^, *p* < 0.01 *vs.* OE-C/EBPβ.

We further introduced the siRNA-resistant C/EBPβ plasmid by synonymous mutation. The results found that transfection of the siRNA-resistant C/EBPβ plasmid (from 24 h before irrradiaiton until 48 h after irradiation) could increase C/EBPβ expression and inhibit PIEZO1 expression. Furthermore, overexpression of C/EBPβ using the siRNA-resistant C/EBPβ plasmid could fully reverse the enhanced PIEZO1 mRNA (Figure 5A) and protein (Figure 5B) expression by si-C/EBPβ. Taken together, these results support that C/EBPβ acts on PIEZO1 promoter to decrease PIEZO1 expression.

## Discussion

**FIGURE 6:**
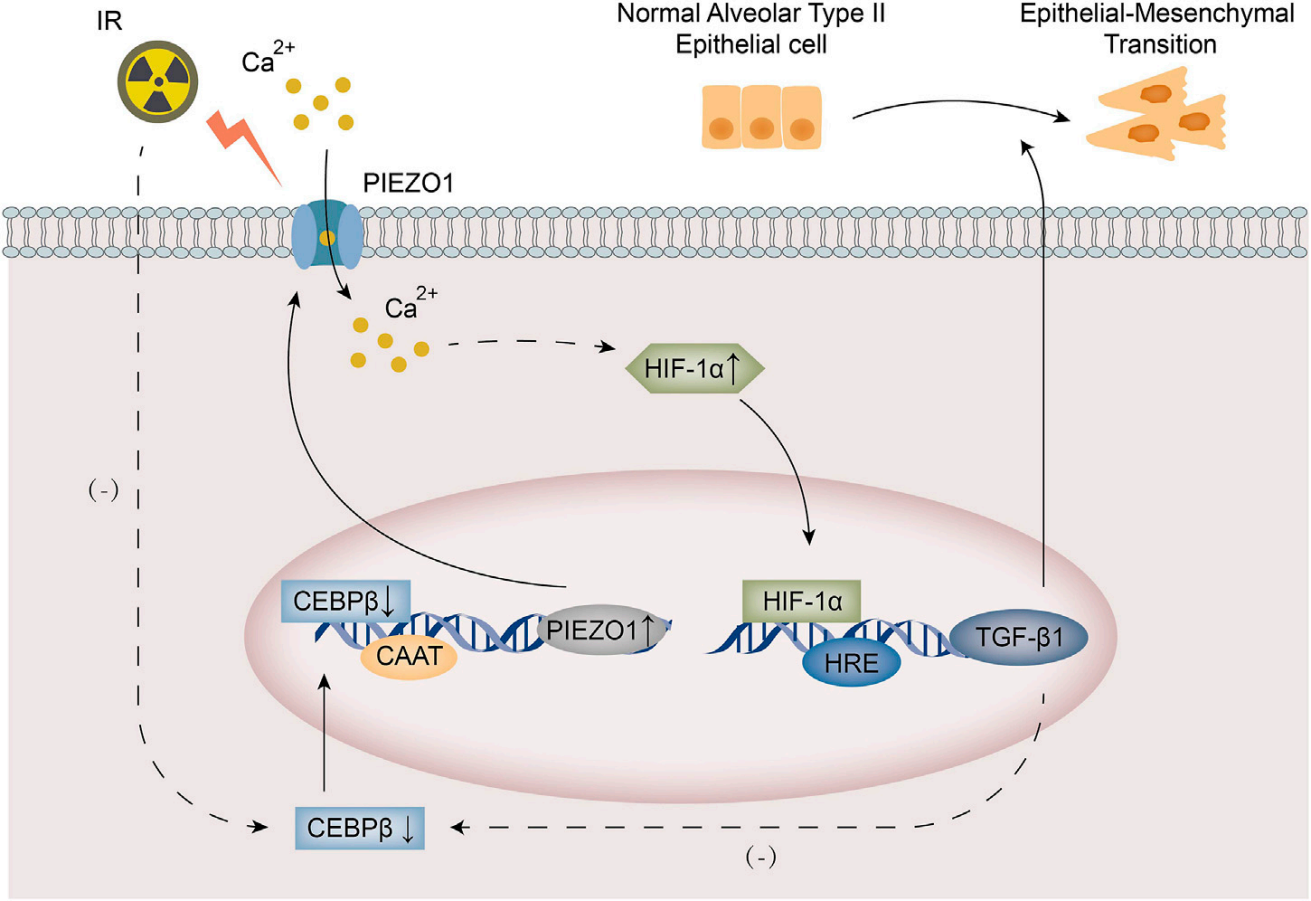
A schematic diagram summarized the mechanism of PIEZO1/Ca^2+^ signaling in radiation-induced epithelial-to-mesenchymal transition (EMT) by forming a positive feedback with TGF-β1. Irradiation leads to increased PIEZO1 expression and decreased C/EBPβ expression in rat alveolar type II epithelial (RLE-6TN) cells. Increased expression of PIEZO1 enhanced Ca^2+^ influx, which activates HIF-1α to induce TGF-β1 expression and following EMT. C/EBPβ expression was inhibited by exogenous TGF-β1. C/EBPβ binds to the promoter region of PIEZO1 to inhibit PIEZO1 expression. The PIEZO1/HIF-1α/TGF-β1/C/EBPβ axis constitutes a positive feedback mechanism.

In this study, we found that PIEZO1 expression was increased in rat alveolar type II epithelial cells after irradiation (IR). Increased expression of PIEZO1 increases intracellular Ca^2+^ concentration, which further increases the TGF-β1 expression through HIF-1α and causes epithelial-to-mesenchymal transition (EMT). The transcription factor C/EBPβ expression was inhibited by irradiation and exogenous TGF-β1. C/EBPβ could bind to the PIEZO1 promoter to increase the expression of PIEZO1. The main findings of current study were summarized in Figure 6.

TGF-β is a major regulator of both physiological (wound healing) and pathological fibrogenesis and is widely considered to be a central pathway of fibrosis (Meng et al., 2016; Gyorfi et al., 2018). Many studies have confirmed that the TGF-β signaling pathway plays an important role in the activation of EMT, causing the process of pulmonary fibrosis. Zavadil et al. (2004) discovered that TGF-β1 can activate Jagged1/Notch signalling through both Smad3-dependent and ERK-dependent mechanisms to initiate EMT. A recent study proved that TGF-β1 secreting M2 macrophages play an important regulatory role in mesenchymal transition of epithelial cells in the lung of irradiated mice, thus contributing to radiation-induced pulmonary fibrosis (Park et al., 2019). In the AT II cells, TGF-β1 has been found to act *via* MAPK and Smad-dependent signaling pathways to induced EMT and pulmonary fibrosis (Guo et al., 2021). Consistent with our findings, we found that the expression of TGF-β1 and the EMT markers N-cadherin, Vimentin, α-SMA, CTGF, and Fibronectin was consistently elevated in rat lung and RLE-6TN cells after 12 Gy ionizing radiation exposure, supporting that 12 Gy ionizing radiation is sufficient to induce AT II EMT. The radiation dosage here is the same as the study by Rube et al., which showed that thoracic irradiation with a dosage of 12 Gy could cause acute (1, 3 and 6 h post irradiation) and long-lasting (2 and 4 weeks after irradiation) increase in TGF-β expression in the AT II cells (Rube et al., 2000).

It has been suggested that PIEZO1 in myeloid cells plays an important role in the process of bleomycin-induced pulmonary fibrosis by promoting autoinflammatory processes (Solis et al., 2019). Recent study also reported that PIEZO1 is expressed and functionally active in cardiac fibroblasts (Stewart and Turner, 2021). PIEZO1-mediated activation of p38α in cardiac fibroblasts and subsequent secretion of IL-6 could be important in the cardiac remodeling process (Blythe et al., 2019). However, whether there is a role of AT II cell PIEZO1 in pulmonary fibrosis, especially radiation-induced EMT and fibrosis is unclear. We found that the expression of PIEZO1 and TGF-β1 was concurrently increased after irradiation in lung tissues and rat alveolar type II epithelial (RLE-6TN) cells. Moreover, knockdown of PIEZO1 decreased TGF-β1 expression induced by irradiation. These results suggest that activation of PIEZO1 may lead to lung epithelial cell EMT through up-regulation of TGF-β1, indicating another source of pro-fibrosis during pulmonary fibrosis.

The activation of PIEZO1 channel leads to calcium influx, which seems to be indispensable for PIEZO1 signal transduction (Coste et al., 2010). Our previous study found that PIEZO1 acts *via* Ca^2+^/calpain signaling to mediate oxygen-glucose deprivation/reoxygenation induced neuron injury (Wang et al., 2019). In urothelial cells, PIEZO1 senses the mechanical stretch stimuli and promote ATP release through increase in cytosolic Ca^2+^ concentrations (Miyamoto et al., 2014). Ca^2+^ is verified regulators of HIF-1 activity (Azimi, 2018). PIEZO1 induces EDN1 expression through Ca^2+^ influx to drive HIF-1α accumulation and inflammation in lung myeloid cells (Solis et al., 2019). In the meantime, HIF-1α signaling has been found to play an essential role in EMT and lung fibrosis (Chen et al., 2019). Ueno et al. (2011) highlighted a crucial role of HIF-1α in mediating the effects of TGF-β1 on PAI-1 expression during bleomycin-induced pulmonary fibrosis. We found that after irradiation or Yoda one exposure, the Ca^2+^ concentration increased, expression of HIF-1α increased, and expression of TGF-β1 increased. PIEZO1 specific siRNA attenuated the Ca^2+^ influx, HIF-1α accumulation as well as TGF-β1 expression caused by irradiation. Moreover, knockdown of HIF-1α partly reversed the EMT-inducing effects of irradiation. Taken together, these results supported that HIF-1α played an essential role in the modulatory effects of PIEZO1 signaling on radiation-induced TGF-β1 expression and EMT.

The expression of C/EBPα, a homologous transcription factor of C/EBPβ, has been found to be inhibited by TGF-β in a SMAD3-dependent way (Lourenco et al., 2020). In NMuMG cells, data suggested that TGF-β1 can also inhibit C/EBPβ expression through the canonical Smad3 pathway (Ramji and Foka, 2002). Similarly, we found that C/EBPβ expression was inhibited by exogenous TGF-β1. C/EBPβ could regulate cell fates including proliferation, differentiation, and senescence through both positive and negative effects on gene expression (Gomis et al., 2006). A recent study found that C/EBPβ knockdown promoted chemoresistance of NPC cells *via* activating SPINK5 (Liu et al., 2021). We also found that decreased C/EBPβ expression was associated with increased PIEZO1 expression. In irradiated cells, we discovered that Ca^2+^ influx was futher increased after TGF-β1 co-culture and the expression of PIEZO1, HIF-1α and TGF-β1 expression were further promoted by exogenous TGF-β1. Therefore, there is a positive feedback between PIEZO1 and TGF-β1. These results are consistent with previous findings showing several different positive feedback networks of TGF-β signaling during fibrosis (K. Zhang et al., 2017). On the underlying mechanisms, we first used bioinformatics to predict whether C/EBPβ binds to the PIEZO1 promoter region. Thereafter, siRNA technology, dual luciferase reporter gene assay and chromatin immunoprecipitation assay (ChIP) were further used to verify the regulatory role of C/EBPβ transcription factor on PIEZO1 expression by binding to the PIEZO1 promoter. Furthermore, overexpression of C/EBPβ using the siRNA-resistant C/EBPβ plasmid could fully reverse the enhanced PIEZO1 expression by C/EBPβ siRN. Taken together, these results support that C/EBPβ acts on PIEZO1 promoter to decrease PIEZO1 expression.

However, there are some limitations in our study. For instance, the specific mechanism by which PIEZO1 is induced by ionizing radiation through C/EBPβ is not fully investigated. Moreover, we did not study the *in vivo* effects of PIEZO1 on RIPI by modulating PIEZO1 expression specifically in the lung epithelial cells, nor the intercellular interaction effects of PIEZO1 signaling. The study could be further strengthened by the use of PIEZO1 knockdown or knockout mice. Some studies also found other fibrosis-related transcription factors such as CREBP1 and YAP can regulate PIEZO1 expression (Hasegawa et al., 2021; Lee et al., 2021). It is interesting to investigate whether these transcription factors play a role in ionizing radiation induced expression of PIEZO1.

In conclusion, our study revealed a critical role of PIEZO1/Ca^2+^ signaling in radiation-induced EMT by forming a positive feedback with TGF-β1. PIEZO1 plays a crosstalk role between TGFβ and EMT, which implicates that PIEZO1 may be a target for the treatment of EMT-related diseases including radiation-induced pulmonary injury (RIPI) and pulmonary fibrosis.

## Data Availability

The original contributions presented in the study are included in the article/Supplementary Material, further inquiries can be directed to the corresponding authors.
