# Supplementary material for: Mechanically Activated Calcium Channel PIEZO1 Modulates Radiation-Induced Epithelial-Mesenchymal Transition by Forming a Positive Feedback With TGF-β1
Source: Front Mol Biosci. 2021 Oct 13;8:725275. doi: 10.3389/fmolb.2021.725275 (PMC8548710; doi:10.3389/fmolb.2021.725275)
Supplement: Supplementary file 1 [file DataSheet1.PDF]

Supplementary Information includes:

- fscans of the original gels (PDF)
- source data of fscans of the original gels (excel)
- flow cytometry data (fcs)

Figure S1

A

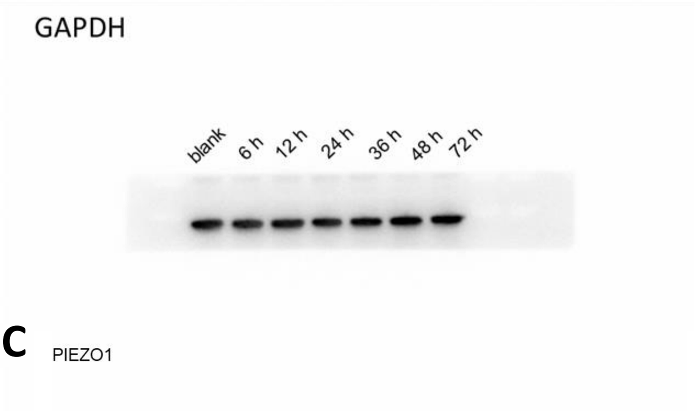

B

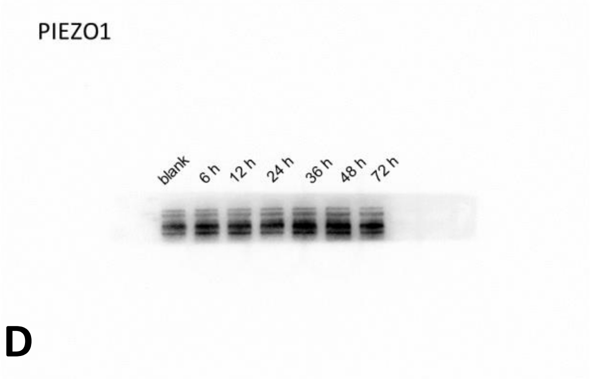

C

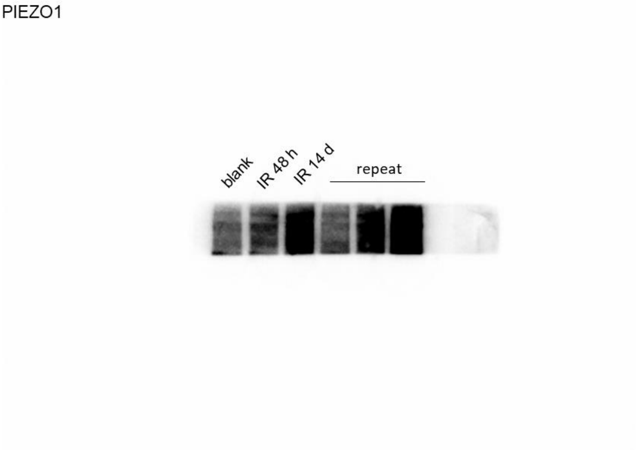

D

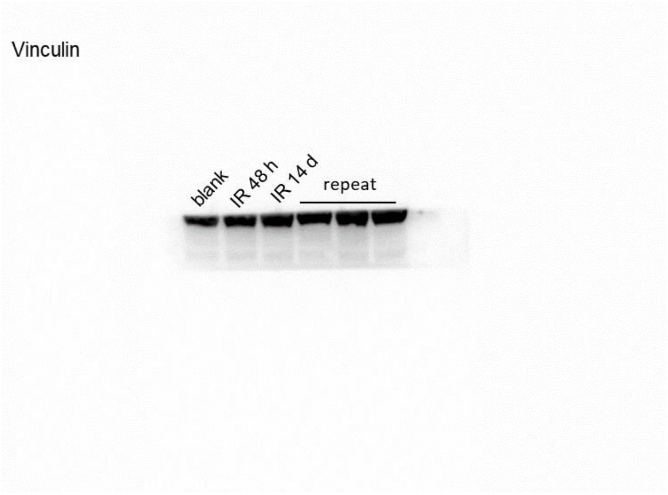

Figure S1 A-B Full scans of the entire original gels displayed in **Figure1 C**; **C-D** Full scans of the entire original gels displayed in **Figure1 D**.

Figure S2

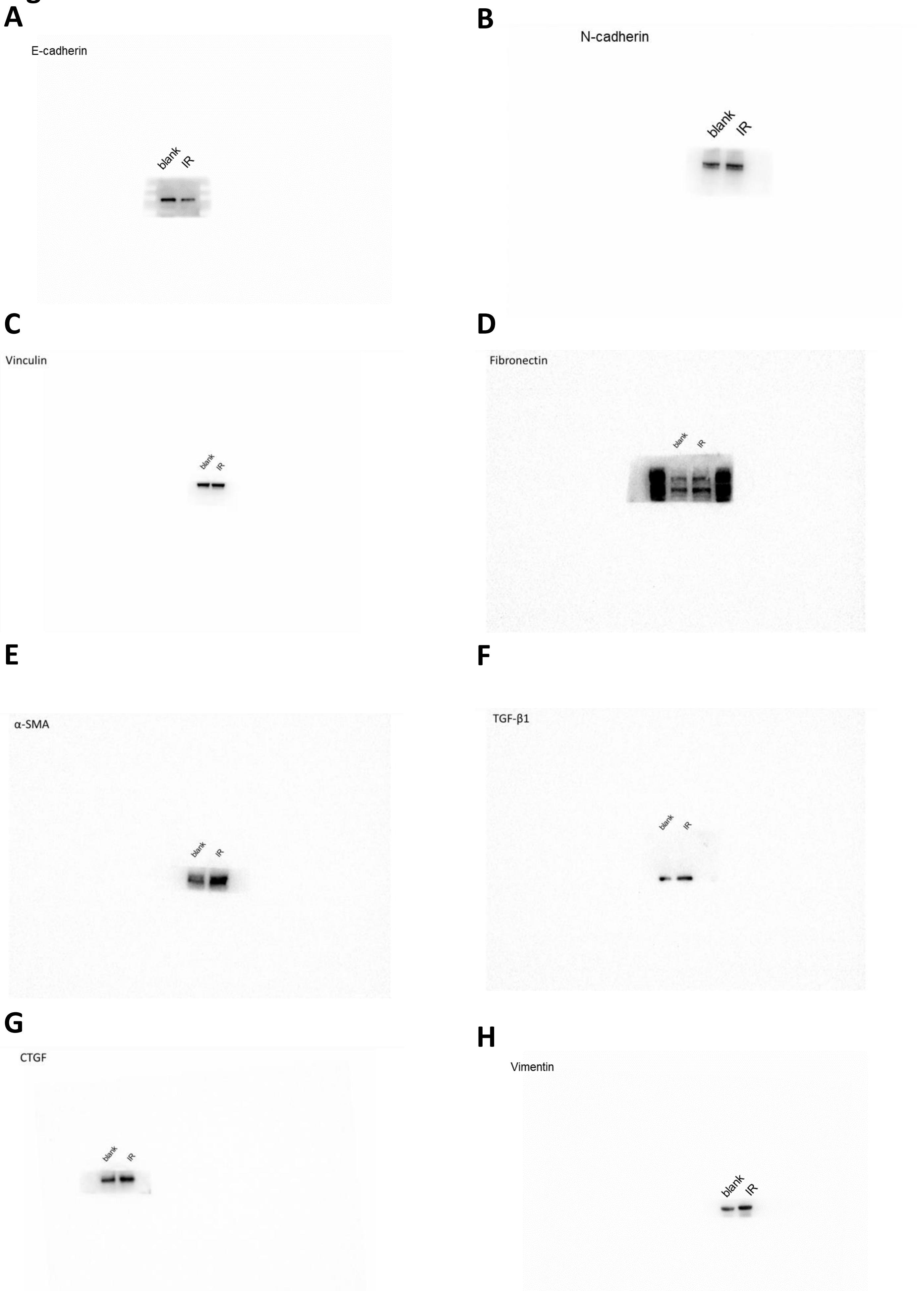

Figure S2 A-H Full scans of the entire original gels displayed in **Figure2 B**.

Figure S3

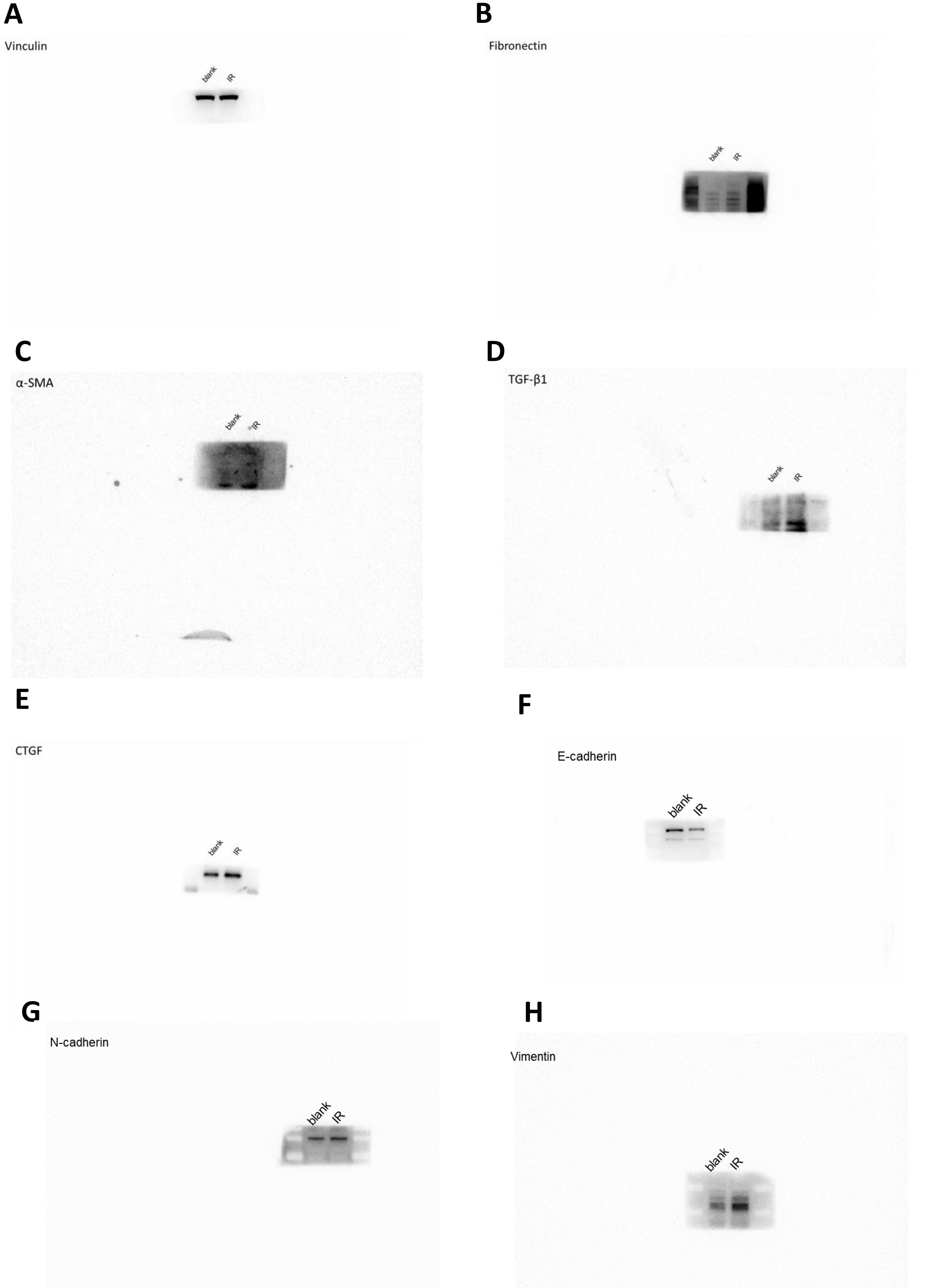

Figure S3 A-H Full scans of the entire original gels displayed in **Figure2 C**.

Figure S4

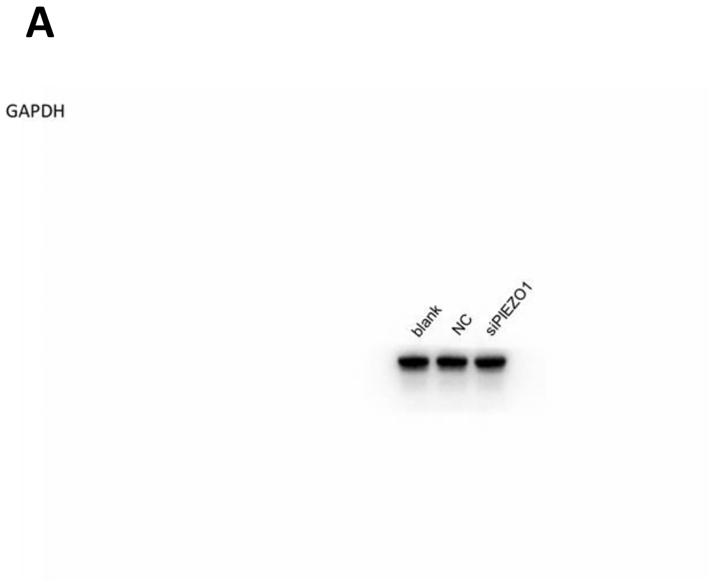

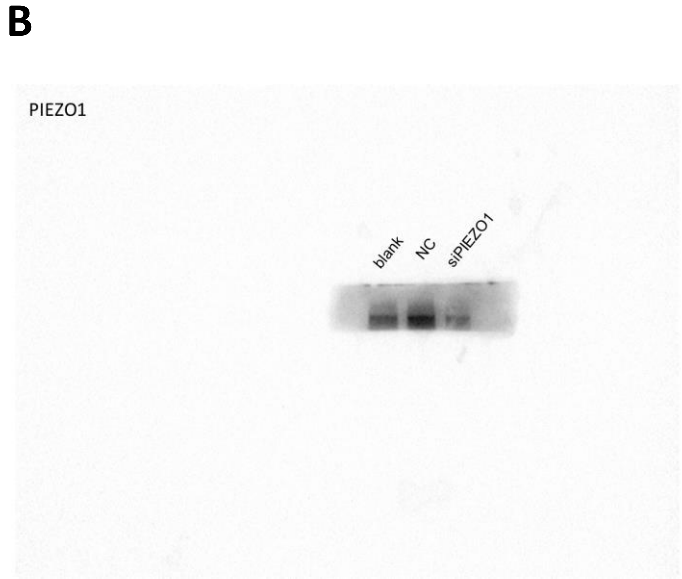

Figure S4 A-B Full scans of the entire original gels displayed in **Figure2 D**.

Figure S5

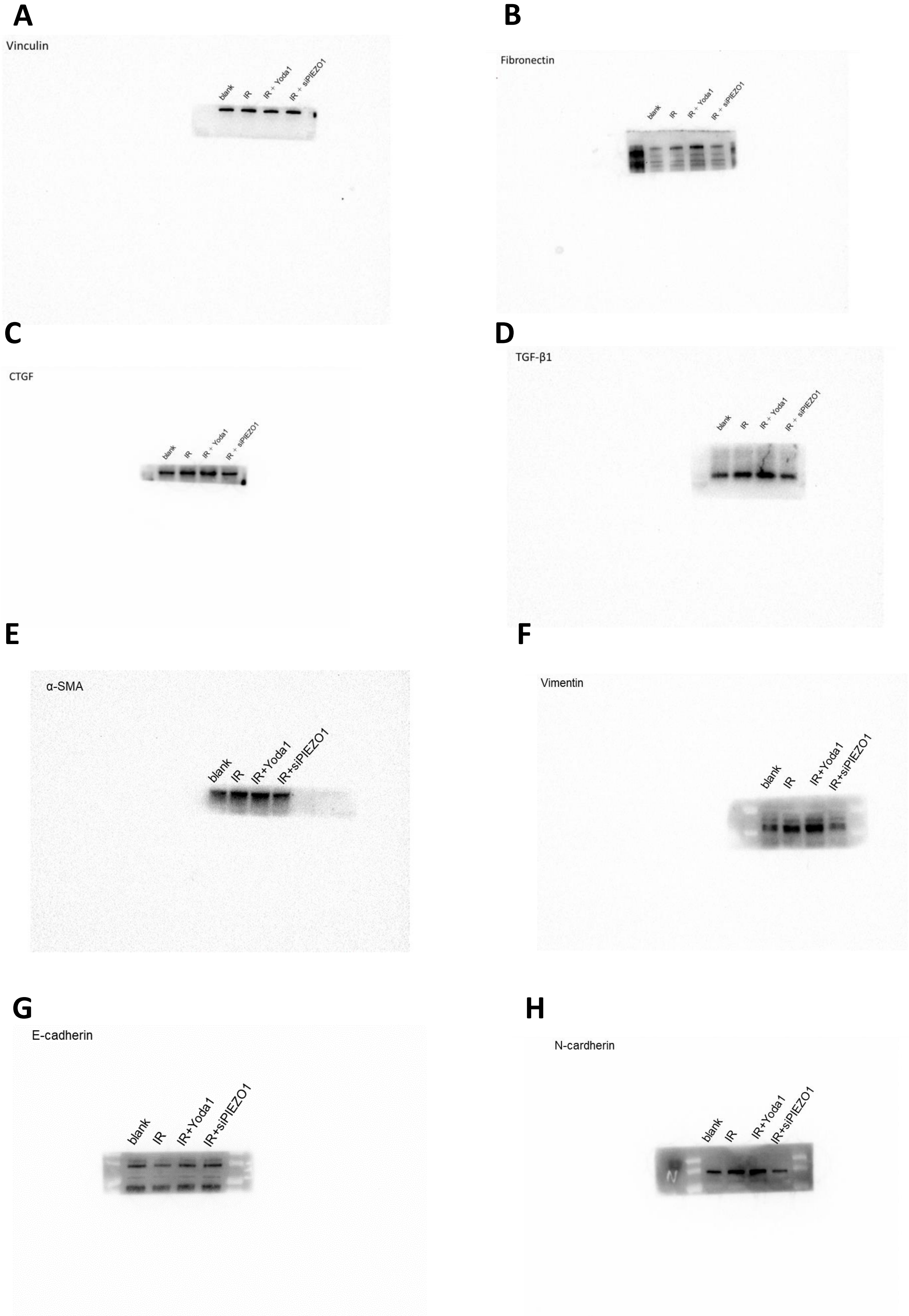

Figure S5 A-H Full scans of the entire original gels displayed in **Figure2 E**.

Figure S6

A

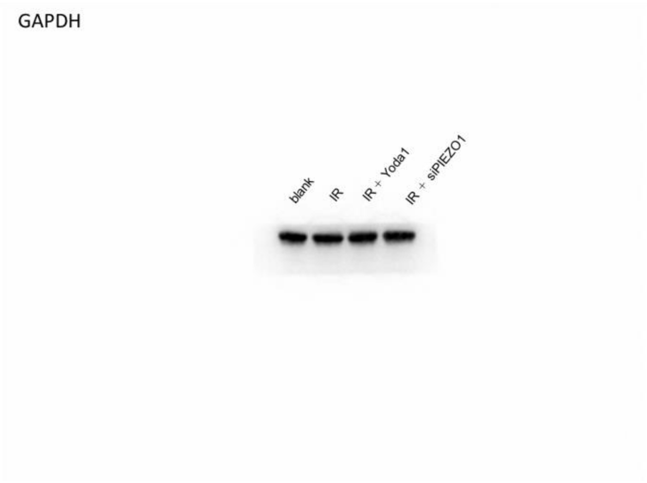

B

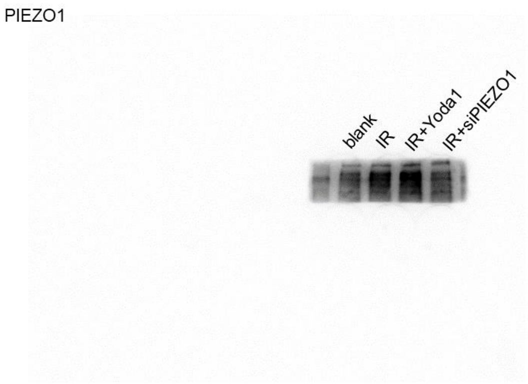

C

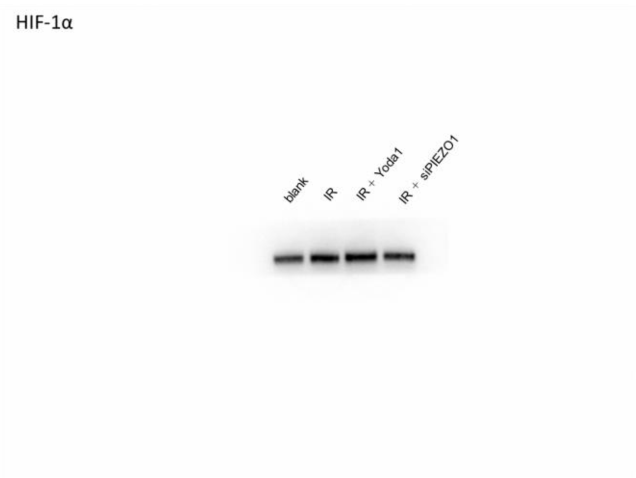

D

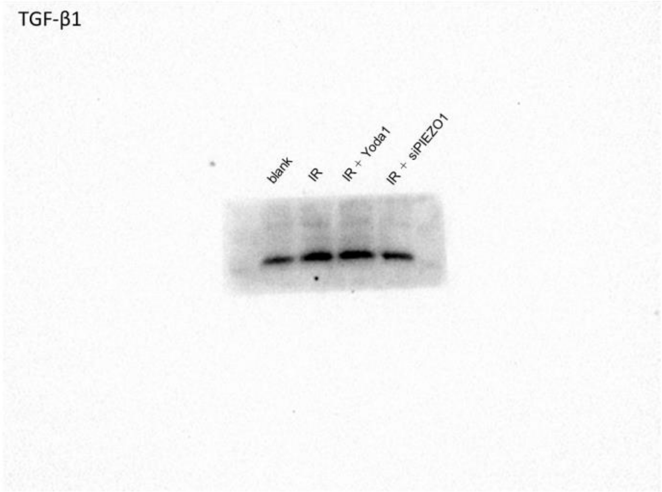

Figure S6 A-D Full scans of the entire original gels displayed in **Figure3 E**.

Figure S7

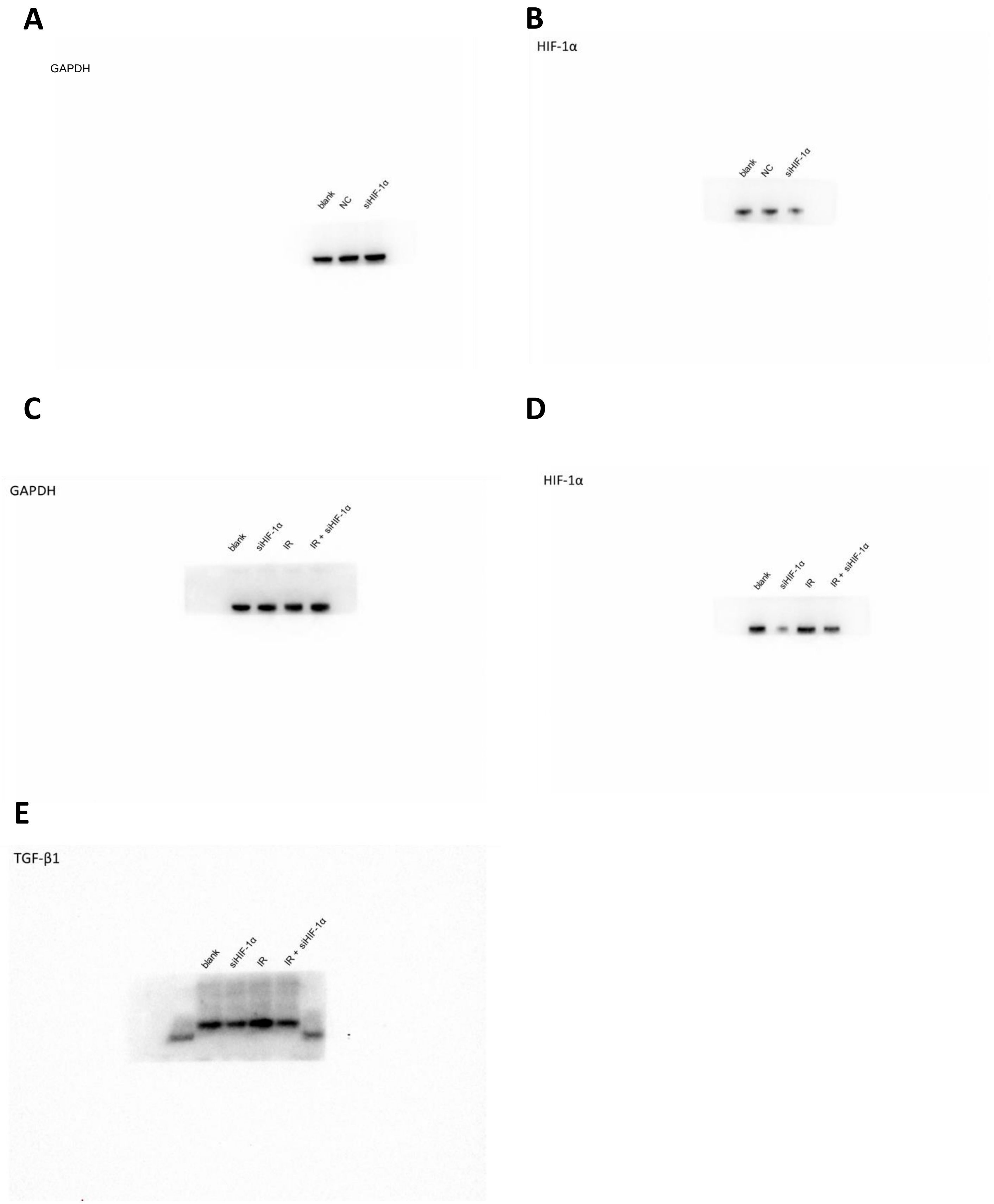

Figure S7 A-B Full scans of the entire original gels displayed in **Figure3 F**; **C-E** Full scans of the entire original gels displayed in **Figure3 H.**

Figure S8

A

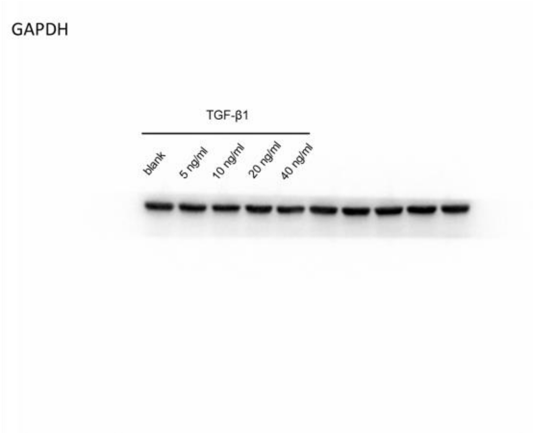

B

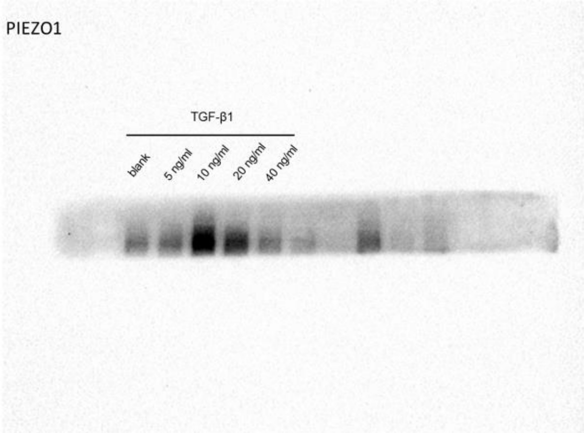

C

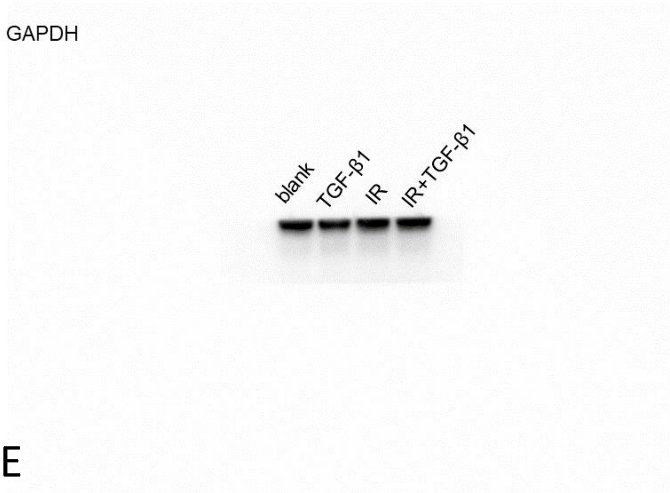

D

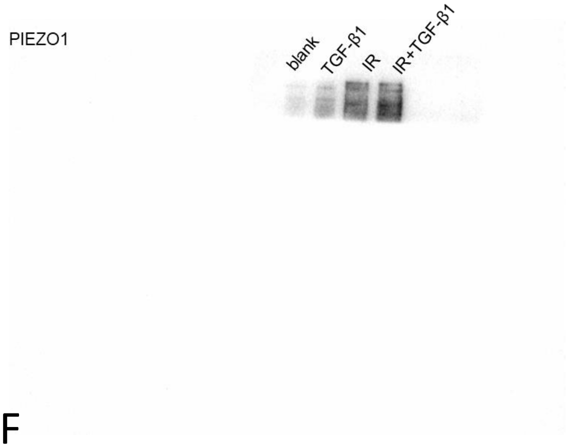

E

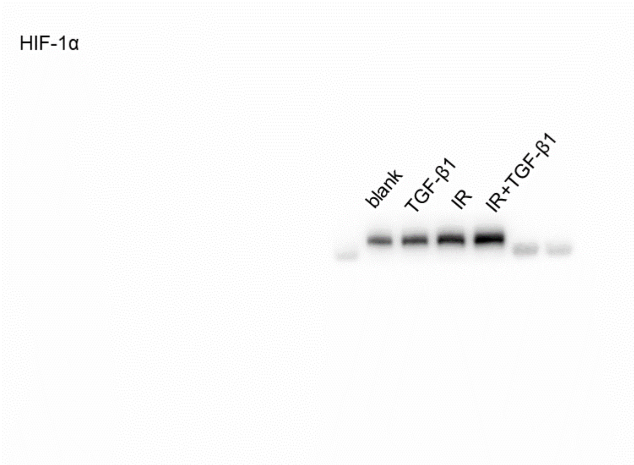

F

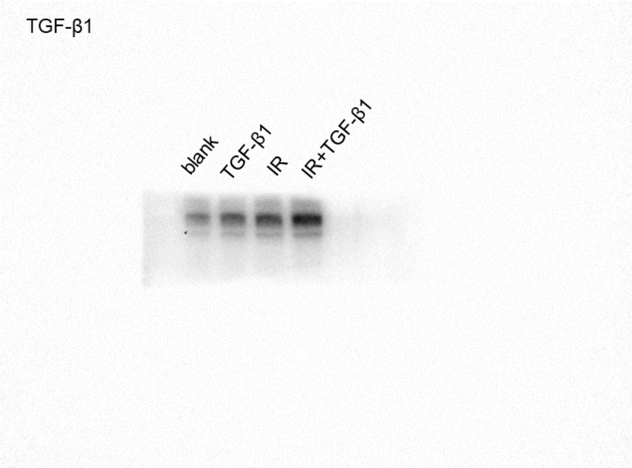

Figure S8 A-B Full scans of the entire original gels displayed in **Figure4 A**; **C-F** Full scans of the entire original gels displayed in **Figure4 B;**

Figure S9

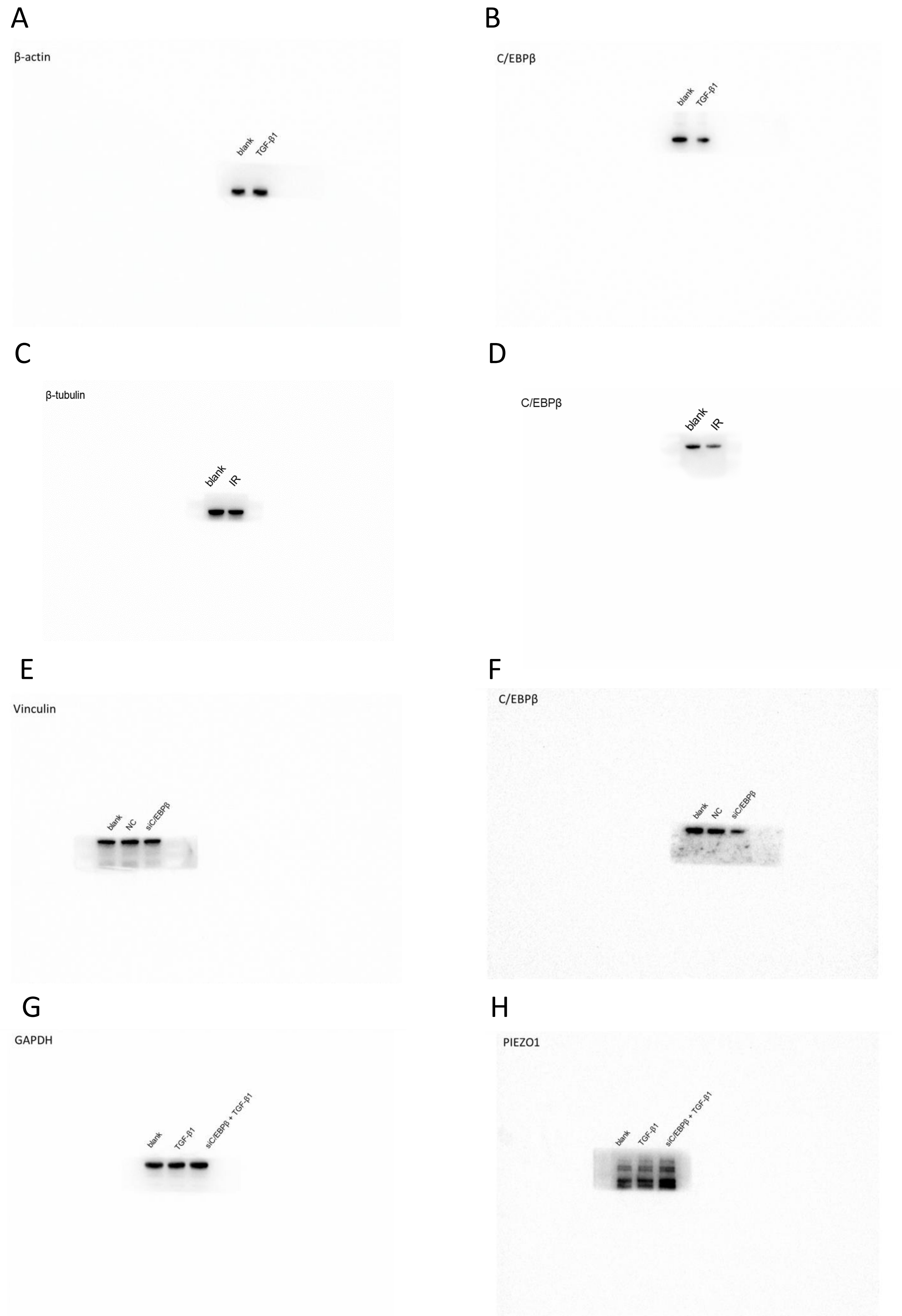
A
β-actin
blank
TGF-β1
B
C/EBPβ
blank
TGF-β1
C
β-tubulin
blank
IR
D
C/EBPβ
blank
IR
E
Vinculin
blank
NC
siC/EBPβ
F
C/EBPβ
blank
NC
siC/EBPβ
G
GAPDH
blank
TGF-β1
siC/EBPβ + TGF-β1
H
PIEZO1
blank
TGF-β1
siC/EBPβ + TGF-β1

Figure S9 A-B Full scans of the entire original gels displayed in **Figure4 D**;
C-D Full scans of the entire original gels displayed in **Figure4 E;**
E-F Full scans of the entire original gels displayed in **Figure4 F;**
G-H Full scans of the entire original gels displayed in **Figure4 G.**

Figure S10

A

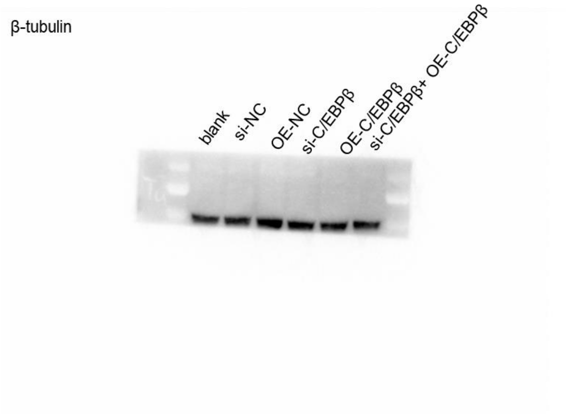

B

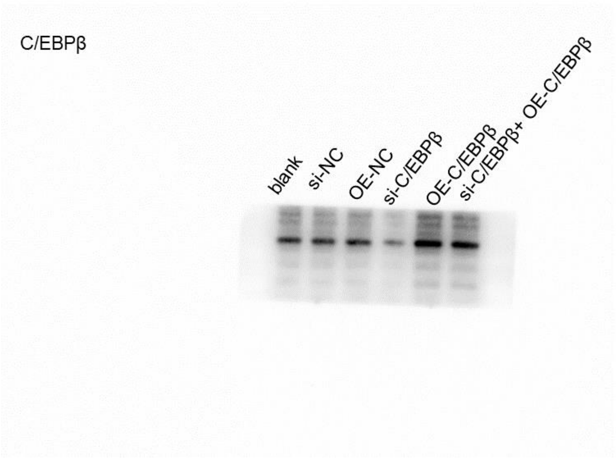

C

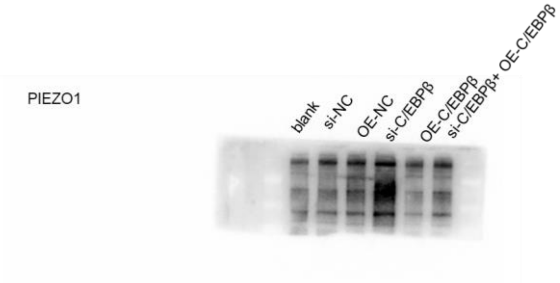

Figure S10 A-C Full scans of the entire original gels displayed in **Figur5 B.**
